# Circadian Timing of Injury-Induced Cell Proliferation in Zebrafish

**DOI:** 10.1371/journal.pone.0034203

**Published:** 2012-03-29

**Authors:** Maria Laura Idda, Elena Kage, Jose Fernando Lopez-Olmeda, Philipp Mracek, Nicholas S. Foulkes, Daniela Vallone

**Affiliations:** Institute of Toxicology and Genetics, Karlsruhe Institute of Technology, Eggenstein, Germany; Vanderbilt University, United States of America

## Abstract

In certain vertebrates such as the zebrafish, most tissues and organs including the heart and central nervous system possess the remarkable ability to regenerate following severe injury. Both spatial and temporal control of cell proliferation and differentiation is essential for the successful repair and re-growth of damaged tissues. Here, using the regenerating adult zebrafish caudal fin as a model, we have demonstrated an involvement of the circadian clock in timing cell proliferation following injury. Using a BrdU incorporation assay with a short labeling period, we reveal high amplitude daily rhythms in S-phase in the epidermal cell layer of the fin under normal conditions. Peak numbers of S-phase cells occur at the end of the light period while lowest levels are observed at the end of the dark period. Remarkably, immediately following amputation the basal level of epidermal cell proliferation increases significantly with kinetics, depending upon the time of day when the amputation is performed. In sharp contrast, we failed to detect circadian rhythms of S-phase in the highly proliferative mesenchymal cells of the blastema. Subsequently, during the entire period of outgrowth of the new fin, elevated, cycling levels of epidermal cell proliferation persist. Thus, our results point to a preferential role for the circadian clock in the timing of epidermal cell proliferation in response to injury.

## Introduction

Cell proliferation plays a key role in the process of tissue regeneration that follows injury. The remarkable ability of most tissues including the heart and central nervous system to completely regenerate upon injury has firmly established the zebrafish (*Danio rerio*) as a powerful model to study tissue regeneration [1], [2], [3], [4], [5]. In particular, the process whereby the amputated caudal fin is able to regenerate in a short time has received significant attention as an experimental model system [6], [7]. The skeleton of the zebrafish caudal fin is composed of alternating segmented bony rays (lepidotrichia) that radiate out from the base of the fin. The rays are separated by inter-ray tissue that is formed primarily by a multilayered epidermis. Each bony segment consists of two concave hemi-rays that surround a core of mesenchymal cells, blood vessels, connective tissue and nerves. Both rays and inter-rays are covered by multiple layers of epithelial cells (Figure S1A) [8], [9], [10]. During the first 24 hours following amputation, wound healing and the formation of the apical epidermal cap (AEC) represent the first key steps towards regeneration. These steps do not involve local increases in cell proliferation [6], [7]. However, the epidermal cells, several segments away from the amputation plane, proliferate and migrate towards the wound [11], [12]. Subsequently, a group of highly proliferative mesenchymal cells near the amputation site form a structure called the blastema (Figure S1B) [11], [13], [14]. The blastema is categorized into regions based in part on cell cycle behaviour: a distal, non- or slow-proliferative area, directly contacting the apical cap, an intensely proliferating proximal region and a more proximal zone where the new cells differentiate into the cell types of the new tissue [6], [13].

Using various approaches including ectopic gene expression, gene knockdown or chemical inhibitors [6], we have now gained considerable insight into the network of signaling events required during outgrowth. Furthermore, it is clear that epithelial-mesenchymal cell interactions at the epidermal cap play an important role in the re-growth and differentiation of the new fin tissue [11], [15], [16], [17]. Thus, spatial control of cell proliferation is of fundamental importance for the correct regeneration of the damaged fin [11], [14]. However, the mechanisms that time the key events of fin regeneration remain unclear. Therefore addressing the contribution of cellular timing mechanisms is a key issue in understanding the regeneration mechanism.

The circadian clock is a conserved cell autonomous timing mechanism whereby organisms anticipate and respond to day-night changes of the environment [18], [19]. Central to the circadian timing system is a pacemaker that oscillates with a period of *circa* 24 hours. Thus, to remain synchronized with the day-night cycle, environmental timing signals (*zeitgebers*) such as light reset this pacemaker via an input pathway. Importantly, clock output pathways subsequently convey this timing information to almost every aspect of physiology in the organism. The zebrafish has been established as an important model system to study various aspects of the circadian timing system [20]. As in other vertebrates, most zebrafish tissues contain independent circadian clocks (so-called peripheral clocks) [21], [22]. While in mammals, light entrainment of peripheral clocks occurs indirectly via the retina and the central clock of the suprachiasmatic nucleus [23], in the zebrafish the peripheral clocks are directly entrained by exposure to light [24], [25]. There is now considerable information concerning the molecular organization of the vertebrate core clock mechanism [18], [20]. Pacemaker function is driven by complex interlocking transcription-translation feedback loops. Transcription factors such as Clock and Bmal activate the transcription of genes belonging to the *Period (Per) and Cryptocrome (Cry)* families via binding to specific promoter sequences termed E-boxes. In turn, the Per and Cry proteins are able to inhibit the transcriptional activation of their own genes. The mechanism also involves additional stabilizing loops [26], [27] as well as complex posttranslational regulation. This additional regulation confers robustness and ensures that the mechanism requires *circa* 24 hours to complete one cycle [18].

One of the key outputs of the clock is the timing of cell cycle progression. Thus, might the circadian clock mechanism contribute to the timing of tissue regeneration in zebrafish? From cyanobacteria to higher vertebrates, there is evidence that the circadian clock gates regulatory steps in DNA synthesis and mitosis [28], [29]. Circadian rhythms of cell cycle have been reported in many vertebrate peripheral tissues included skin, intestine, bone marrow, liver, gut, heart etc. [29], [30], [31], [32], [33]. The genes *wee1*, *c-myc* and *p21* appear to represent key clock regulatory targets in this process [33], [34], [35], [36]. The gene encoding the *wee1* kinase, a regulator of the G2/M checkpoint, is clock regulated due to the presence of E-box elements in its promoter [34], [35]. Its robust circadian oscillation is lost in Cry- and Clock- deficient mice resulting in impairment of hepatocyte proliferation [34]. Also the cyclin-dependent kinase inhibitor *p21* that inhibits passage through the G1/S transition is rhythmically expressed in mouse peripheral organs and is regulated by core clock elements [36], [37].

Previously, we have reported that in 5-days-old zebrafish larvae, the circadian clock generates daily S-phase rhythms in various tissues by a cell-autonomous mechanism [31]. In addition, we revealed that this mechanism operates in concert with systemic signals of which glucocorticoids are important players [38].

Here we show that circadian rhythms of the cell cycle represent a hallmark of the cell proliferation that occurs during fin regeneration. Interestingly, circadian rhythms of the cell cycle are restricted to the epidermis and notably absent from the blastema. In addition our data reveal a strong, time-of-day dependence for key early cellular responses to injury.

## Results

### High amplitude circadian cell cycle rhythms exist in adult zebrafish fins

Much of our previous work investigating the zebrafish circadian clock has been performed in embryonic cell lines, embryos or larvae [25], [31], [38], [39], [40]. Thus, as a first step we wished to confirm that like most other zebrafish tissues the adult caudal fin possesses a light-regulated circadian clock.

**Figure 1:**
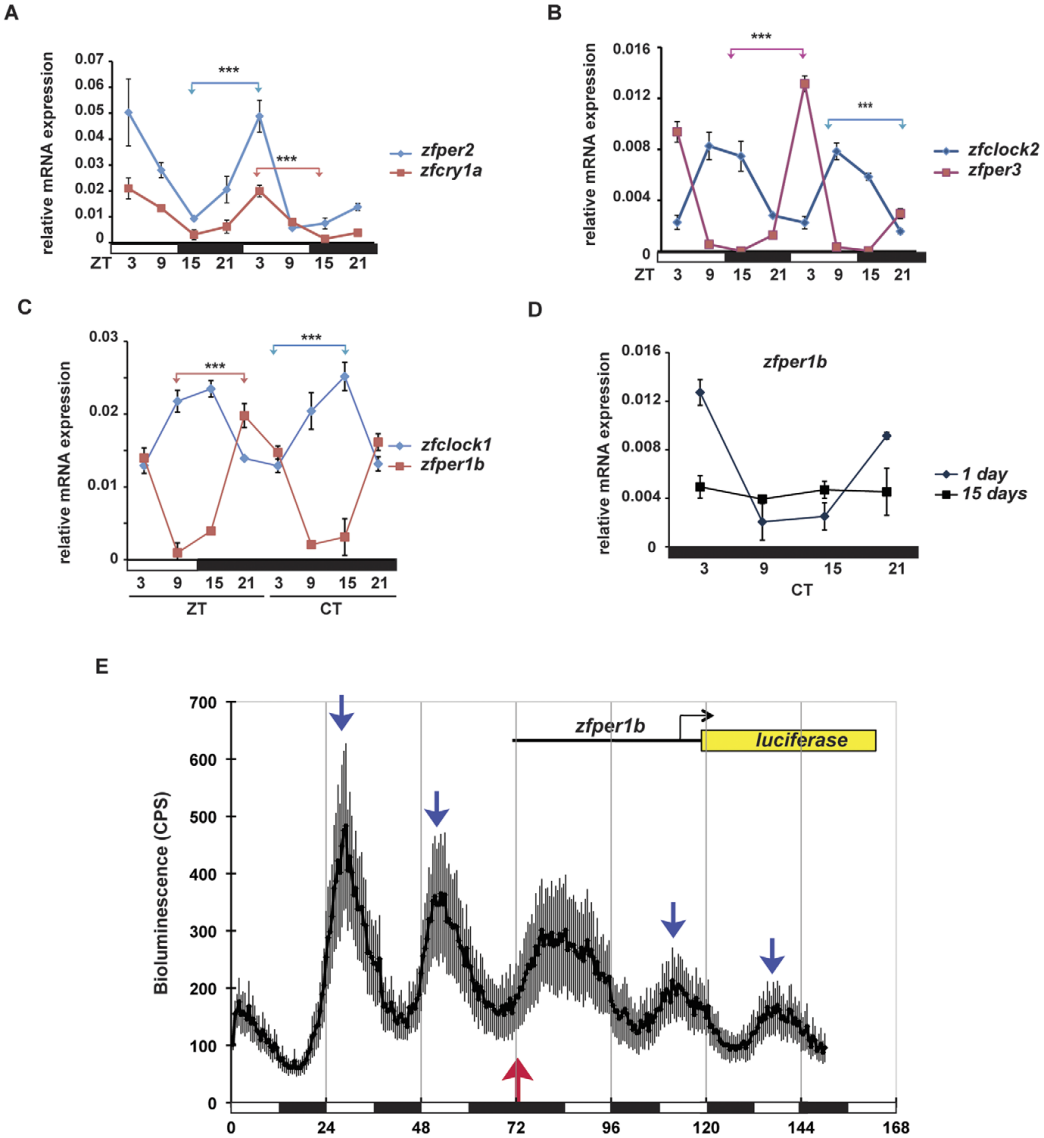
Rhythmic clock gene expression in zebrafish caudal fins. (A–D) Quantitative RT-PCR analysis of clock gene expression in the adult caudal fin of zebrafish. (A–C) All genes show statistically significant differences between peak and trough values (Bonferroni's *post hoc* test p<0.0001) under light-dark (LD) conditions. (C) *zfper1b and zfclock1* rhythmic expression persists on the first day in constant darkness DD. (D) Lack of oscillation of *zfper1b* after 15 days in DD, free running conditions, compared with the rhythmic expression still observed after 1 day under DD conditions. The time of each sample is indicated either as zeitgeber time (ZT, where ZT0 is defined as lights on and ZT12, lights off) under LD cycle conditions (A–C) or circadian time (CT) under constant darkness (C–D). The results of statistical analysis are indicated above each graph by asterisks and colour-coded horizontal “brackets” drawn between the peak and trough values analysed. Black and white bars beneath each panel indicate the dark and light periods of the lighting regimes. Data for all genes were subjected to Cosinor analysis to test for the presence or absence of 24-h rhythmicity (see Table S1, Figure S2). For each time point a pool with a minimum of n = 5 fins were used. In each panel, points are plotted as means of three independent experiments +/− SEM. (E) Mean levels of bioluminescence measured from an *in vivo* luciferase assay of primary zebrafish caudal fin cell cultures. Cells were transiently transfected with the clock regulated reporter construct *zfper1b-luc* [25] and then assayed in real time while being exposed to various lighting regimes. On the X-axis is plotted the assay time (hours) from the start of the experiment. Blue arrows indicate the daily peaks of bioluminescence while a red arrow denotes the point where the phase of the LD cycle was reversed (LD to DL). Bioluminescence levels were plotted as means +/− SEM from three independent fin primary cultures.

We characterized the expression of a subset of clock genes in this tissue upon exposure of adult zebrafish to 24 hours light-dark (LD) cycles as well as to constant darkness (DD) and constant light (LL) conditions. The expression of *zfclock1*, *zfclock2*, *zfcry1a*, *zfper2*, *zfper1b and zfper3* mRNAs oscillate in a daily manner under LD conditions (Figure 1A–C, Figure S2 and Table S1). Furthermore, as predicted for regulation by a peripheral circadian clock mechanism, rhythmic expression of *zfclock1 and zfper1b* persists during the first and second day in DD and LL (Figure 1C,D, Figure S2 and Table S1) but is subsequently absent after 15 days in constant conditions (Cosinor p = 0.31) (Figure 1D and Table S1). We next verified that this circadian clock mechanism is also directly light entrainable. We transfected a primary cell culture prepared from dissociated caudal fins with a clock regulated luciferase reporter construct (*zfper1b:luc*) [25]. Under LD conditions, we observed robust rhythmicity of bioluminescence and reversal of the light cycle (DL) resulted in a 12 hours shift in the phase of this rhythm (Figure 1E). Thus, together these data support the existence of a light entrainable circadian clock in the adult zebrafish caudal fin.

**Figure 2:**
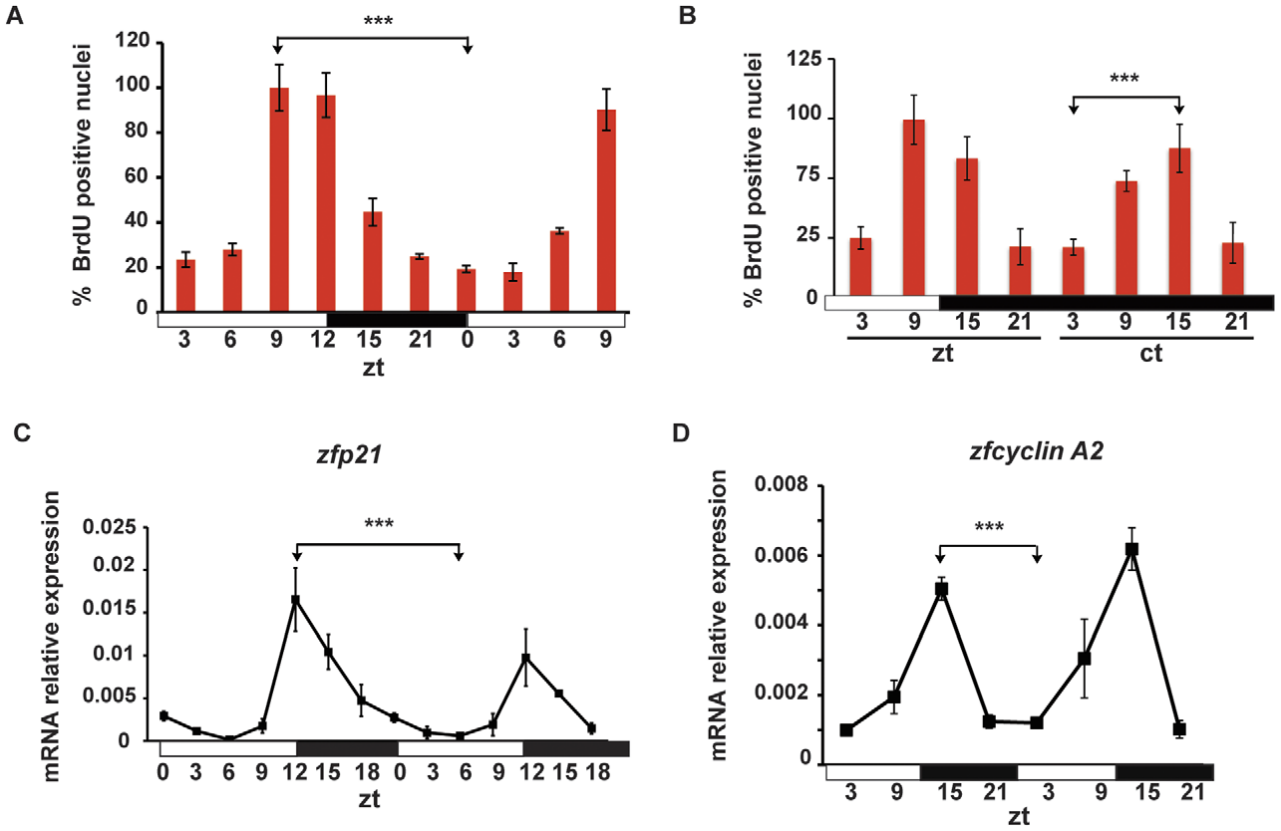
Circadian rhythms of S-phase in zebrafish fins. (A) Numbers of BrdU positive nuclei in adult caudal fins oscillate under LD cycle conditions and (B) during the first day of DD following transfer from LD. On the Y-axis is plotted the % of the BrdU positive nuclei with respect to the peak points. (C, D) Quantitative RT-PCR analysis of *zfp21* and *zfcyclin A2* expression during 2 days of exposure to LD cycles. In each panel, the time of each sample is indicated either as zeitgeber time (ZT) (A, B, C, D) or circadian time (CT) (B). In each panel, each point is plotted as the mean +/− SEM of three independent experiments, each including a minimum of n = 4 fins per point. The results of statistical analysis are indicated above each graph by asterisks (Bonferroni's *post hoc* test p<0.0001) and horizontal “brackets” drawn between the peak and trough values analyzed. White and black bars below indicate the light and dark periods. All the data were subjected to Cosinor analysis to test for the presence or absence of 24-h rhythmicity (see Table S1, Figure S2).

Previously we reported the presence in zebrafish larvae of a daily rhythm of DNA synthesis (S-phase) in different tissues generated by cell autonomous circadian clock mechanisms and systemic signals [31], [38]. Does rhythmic S-phase also exist in the adult caudal fin? We chose to monitor the number of cell nuclei that have entered S-phase at different times of the light- and dark-periods. We used a BrdU-incorporation assay that has been extensively employed in previous studies documenting circadian rhythms of cell cycle [31], [34]. Current published methods for applying this assay using adult zebrafish involve intraperitoneal injection or addition of BrdU to the fish water and then subsequent analysis after relatively long time periods of incubation with BrdU (for a minimum of 6–7 hours [11], [41]). For detecting circadian rhythms of the cell cycle, such experimental approaches lack sufficient resolution. Therefore, as in our previous larval studies [31], [38], we incubated the fish in BrdU–containing water for a very short period (15 minutes) and then immediately fixed the tissue for subsequent analysis. In this way, each timepoint represents labeling during a well-defined, short period. We performed a whole-mount BrdU-incorporation assay using the fins of adult fish maintained under LD cycles, DD and LL conditions. A high amplitude daily rhythm in the numbers of S-phase nuclei was observed in this tissue (Figure 2A, S2 and Table S1), predominantly in the epidermis within the inter-ray regions (Figure S3A) with highest levels during the light – dark transition (ZT9-15) (zeitgeber time, where ZT0 is defined as lights on) and lowest levels at the dark – light transition (ZT 21-3) (Figure 2A). This oscillation persists during the first day in DD (Figure 2B, S2 and Table S1) but disappears after the fish have been maintained for 15 days in DD or LL (Cosinor p = 0.718 and p = 0.319 respectively) (Figure S3B and Table S1). Consistent with these high amplitude S-phase rhythms, we encountered rhythmic mRNA expression for the regulator of the G1/S cell cycle transition *zfp21* and *zfcyclinA2* that regulates both G1/S and G2/M transitions (Figure 2C,D, S2 and Table S1). These data confirm the existence of circadian cell cycle rhythms in the fin, the timing of which closely resemble the previously documented rhythms in zebrafish larvae [31].

**Figure 3:**
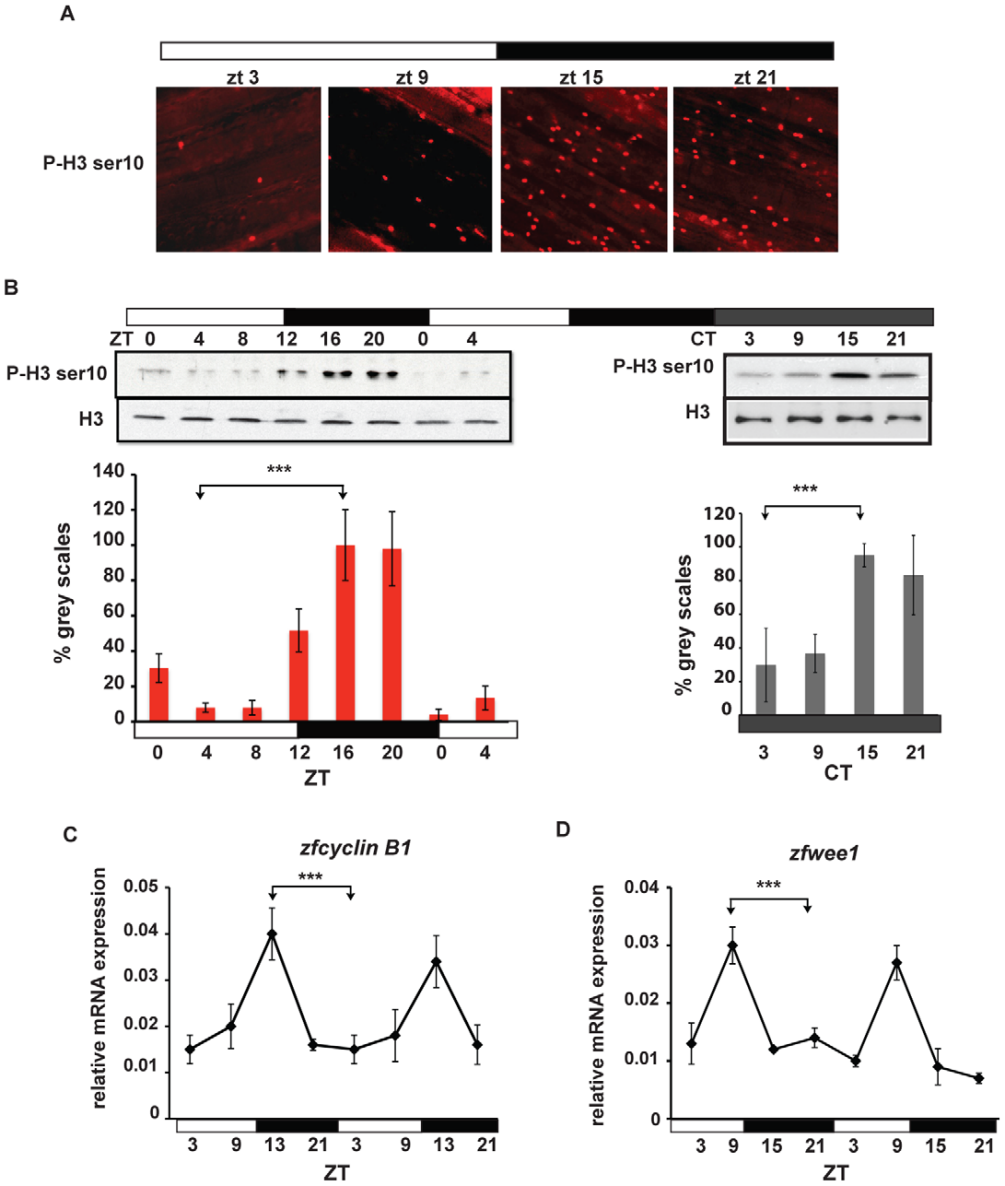
Circadian rhythms of M-phase in zebrafish fins. (A) Immunofluorescence of representative adult caudal fins stained using a phospho Histone H3 Ser 10 (P-H3 ser10) primary antibody at different zeitgeber times. (B) Western blot analysis and quantification of fin whole protein extracts in LD (left panel) and after 1 day in DD (right panel) using Histone H3 (H3) and P-H3 ser10 antibodies. On the Y-axis is plotted the values of grey-scale quantification using Scion Image software and calculated as % relative to the peak timepoint (ZT16 and CT15 respectively). (C,D) Quantitative RT-PCR analysis of two mitotic markers: *zfcyclin B1* (C) and *zfwee1* (D). In all experiments, the time points are indicated either as zeitgeber time (ZT) or circadian time (CT). White and black bars below each panel indicate the corresponding lighting conditions. Each experiment was performed in triplicate with a minimum of 4 fins (n = 4) for each timepoint. Data are plotted as means +/− SEM of three independent experiments. The results of statistical analysis are indicated above each graph by asterisks (Bonferroni's *post hoc* test p<0.0001) and horizontal “brackets” drawn between the peak and trough values analyzed. All the quantitative data were subjected to Cosinor analysis to test for the presence or absence of 24-h rhythmicity (see Table S1, Figure S2).

We confirmed these data by testing whether circadian rhythms of M-phase were also present in this tissue [34]. Using immunofluorescence and Western-blot assays, we quantified the number of cells expressing Histone H3 phosphorylated at ser10 (P-H3 ser10), as an M-phase marker [42], [43], in the caudal fins of fish entrained by LD cycles. We reveal an oscillation in the number of P-H3 ser10 positive cells with highest levels during the middle of the dark period (ZT16 – 20) and lowest levels during the middle of the light period (ZT4 – 8) (Figure 3A,B left hand panel, S2 and Table S1). Furthermore, this rhythm persists after transfer to DD conditions (Figure 3B right hand panel, S2 and Table S1). Consistent with this M-phase rhythm, mRNA expression of the mitotic marker zf*cyclin B1* and the G2/M checkpoint kinase *zfwee1* oscillate in a daily manner in whole fin RNA extracts (Figure 3C,D, S2 and Table S1. Thus, we conclude that in the zebrafish fin the circadian clock robustly regulates cell cycle progression.

### Amputation increases levels of circadian cell proliferation

**Figure 4:**
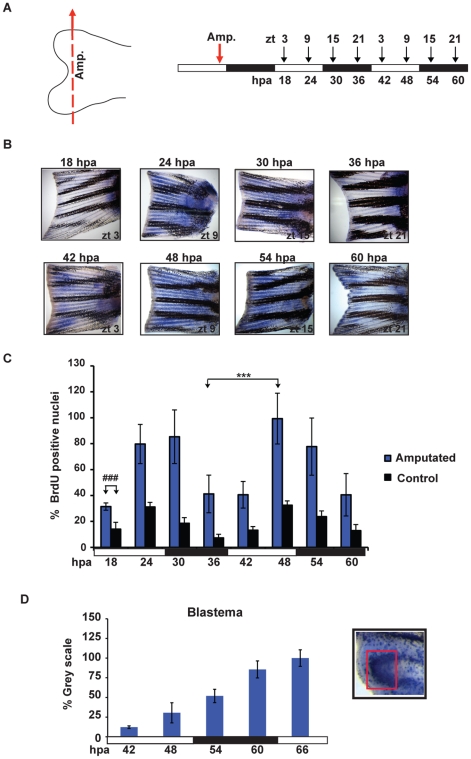
Amputation differentially induces cell proliferation. (A) Schematic representation of the experimental plan showing the amputation site (left panel), the times of sampling (ZT, right panel,,above) and the corresponding hours post amputation (hpa, right panel, below). Amputation was performed before the light-dark transition (ZT 9, red arrow) and fins were subsequently harvested every 6 hours (starting from 18 hpa). Each time point was collected following a 15 minutes incubation with BrdU. (B) Representative fins stained for BrdU incorporation at the time points indicated in panel A. (C) Quantification of the number of S-phase nuclei in amputated fins (blue bars) and non-amputated controls (black bars). On the Y-axis is plotted the % of BrdU positive nuclei with respect to the sample with the largest value (amputated, 48 hpa). The result of statistical analysis of the peak and trough values for the amputated fins is indicated by asterisks (Bonferroni's *post hoc* test p<0.0001) and horizontal “brackets” above the graph. Furthermore, statistically significant differences observed at each time point between the amputated and control non-amputated fins are indicated for simplicity, by the symbol “#” and a bracket above only the first time point (18 hpa) (Bonferroni's *post hoc* test p<0.0001, ###). (D) Quantification of the level of BrdU staining at the ray tips in the blastema region using Scion Image software. Inset panel (D): magnified view of the fin ray tips region stained for BrdU incorporation. A red square delimits the area that has been quantified. White and black bars below each panel indicate the light and dark periods. All the quantitative data were subjected to Cosinor analysis to test for the presence or absence of 24-h rhythmicity (see Table S1, Figure S2). Each time point represents the mean values calculated for each fin ray in a total of n = 4 to 6 fins and expressed as % of the grey scale value.

Does the clock-regulated progression of the cell cycle persist following fin amputation? We assayed the number of BrdU-positive nuclei in the caudal fins of fish maintained under LD cycles and amputated at the end of the light phase (ZT9). After 15 minutes of BrdU incubation, we harvested the injured fins at different ZT times during the subsequent 60 hours and on the 7^th^ day post amputation (7 dpa) (Figure 4 and S4 respectively). Our results show that circadian rhythms of S-phase are retained over the fin during the first 60 hours as well as at 7 days following amputation (Figure 4B,C, S2, S4 and Table S1). The phase of the rhythms matches those of the non-amputated fin controls (see also Figure 2A). Interestingly, from 18 hours post amputation (18 hpa) we observed a striking increase in the overall levels of BrdU-positive cells (two-way ANOVA p<0.0001) (Figure 4C). This dramatic increase in clock-regulated cell proliferation affects the entire fin area (Figure 4B). Importantly, this increase occurs well before the formation of the blastema, which is considered to be the primary site of active cell proliferation following amputation [6], [7]. Next we specifically analysed the timing of cell proliferation in the blastema itself. Consistent with previous reports, we observed an increase in BrdU positive nuclei in the blastema region visible from approximately 42–48 hpa (Figure 4D). However, in contrast to the situation over the remainder of the fin, the high level of cell proliferation within the blastema region shows no evidence of circadian rhythmicity (Cosinor p = 0.72) (Figure 4D and Table S1).

**Figure 5:**
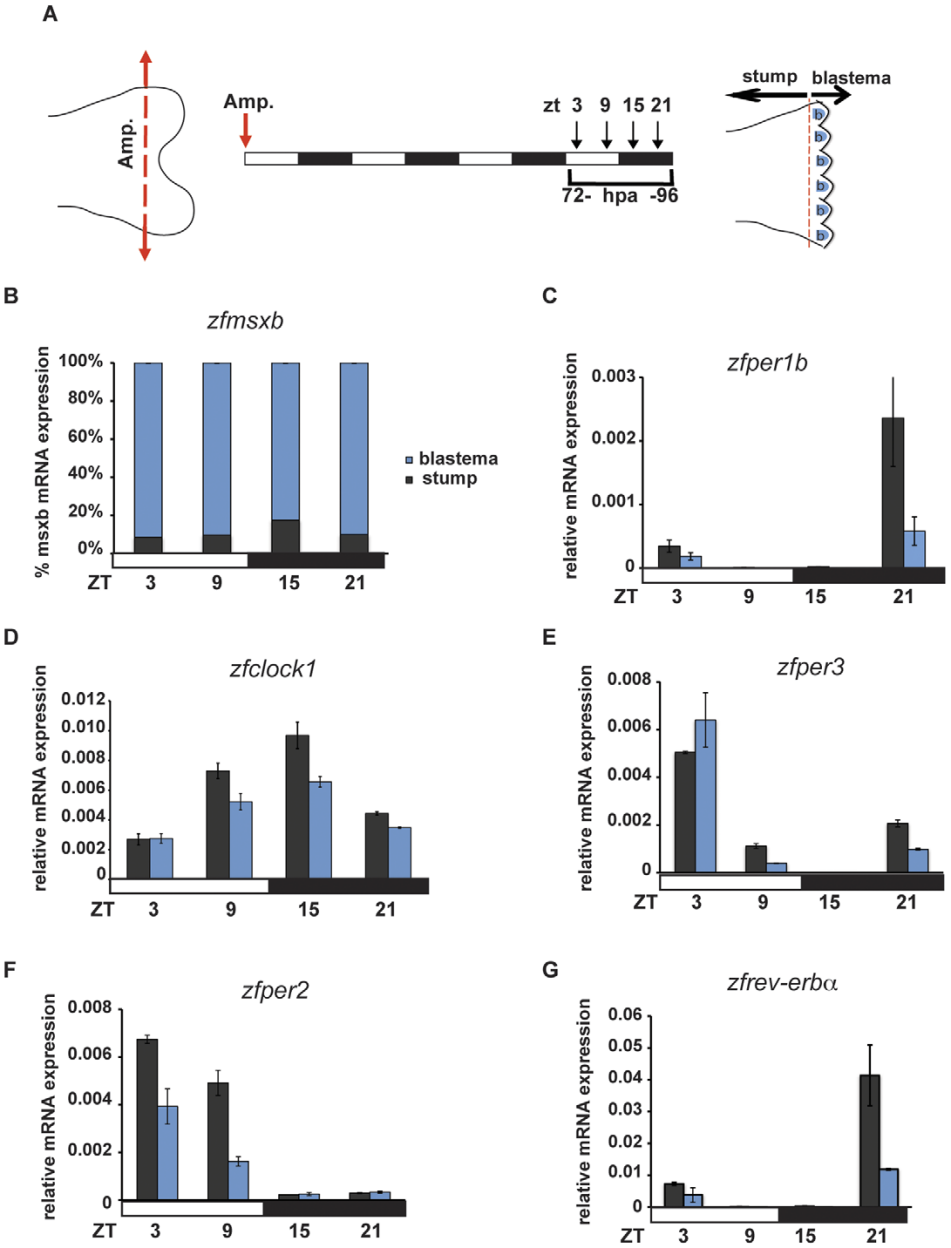
Rhythmic clock gene expression in the blastema region following amputation. (A) Schematic representation of the experimental plan showing the amputation site (left section), the times of sampling (ZT and hpa) with respect to the lighting conditions (central section) and the location of the stump and blastema (b) regions analyzed (right section). Amputation was performed at the dark-light transition (ZT 0, red arrow). (B) Quantitative RT-PCR analysis of a blastema marker (*zfmsxb*) in the stump and blastema regions of amputated fins used as a control for the enrichment of blastema cells in the blastema samples. (C–G) Quantitative RT-PCR analysis of clock genes expression in the stump (black bars) and blastema (blue bars) regions of amputated fins. Each experiment was performed in triplicate with a minimum of 6 fins (n = 6) pooled together for each timepoint. Cosinor analysis of the clock gene expression in the stump as well in the blastema region shows 24-h rhythmicity (see Table S1, Figure S2). Black and white bars beneath each panel indicate the dark and light periods of the lighting regimes.

Does this finding reflect a lack of circadian clock activity within the blastema region? To address this question, we directly analysed clock gene expression in the blastema region and fin stump control between 72 and 96 hpa (Figure 5). As a control we initially confirmed high levels of *zfmsxb* mRNA (a blastema marker [13]) in the dissected blastema region compared with the stump extracts (Figure 5B). Rhythmic expression was observed for all clock genes analysed in both regions (Figure 5C–G, S2 and Table S1) with some reduction in overall expression levels in the blastema compared with the remainder of the fin (Figure 5C–G). Together, these results indicate that the lack of circadian cell cycle rhythms in the blastema is not due to the absence of a circadian clock in this region.

### The circadian increase in cell proliferation is injury-related

**Figure 6:**
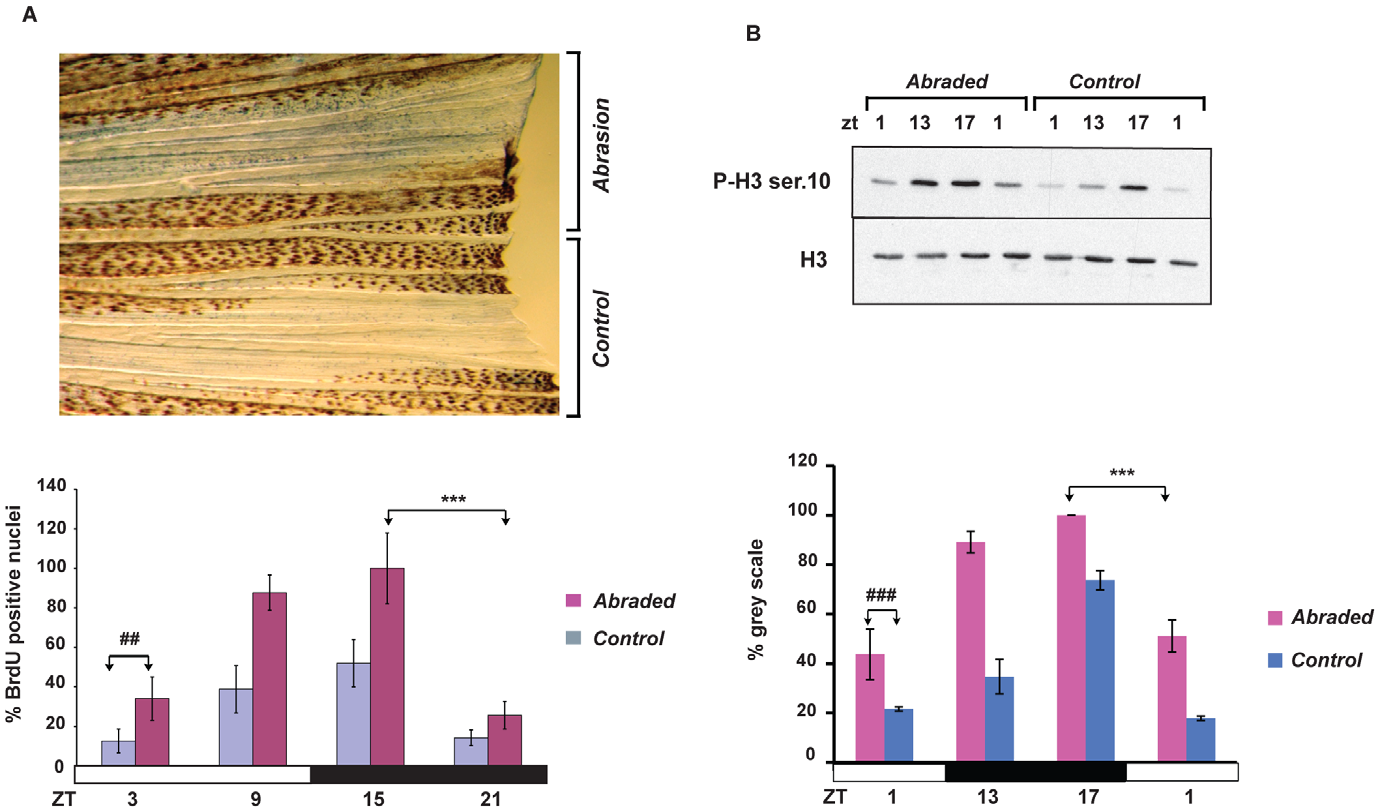
Mechanical abrasion increases circadian cell proliferation. (A), Upper section: Representative image of BrdU-stained caudal fin 48 hours after half of its surface was abraded. The remaining, non-treated half of the fin served as an internal control. Lower section: The results of quantification of the number of BrdU positive nuclei measured each 6 hours during one 24 hours period between 24 and 48 hours following abrasion performed at ZT3. On the Y-axis is plotted the % of BrdU positive nuclei with respect to the largest value (ZT15, abraded). (B) Western blot analysis using P-H3 Ser 10 and H3 antibodies and its quantification (below) of whole protein extracts prepared from the abraded and non-abraded (control) sections of fins. On the Y-axis is plotted the % of grey scale with respect to the highest value (ZT17, abraded). The precise times of sample preparation are indicated by ZT times. Each time point represents the mean value +/− SEM calculated for a minimum of n = 6 fish. The results of statistical analysis of the peak and trough values for the abraded fins are indicated by asterisks (Bonferroni's *post hoc* test p<0.0001) and horizontal “brackets” above the graphs (A and B). Furthermore, statistically significant differences observed at each time point between the abraded and non-abraded control fins are indicated for simplicity, by the symbol “#” and a bracket above only the first time point (panel A, Bonferroni's *post hoc* test p<0.001 and panel B, Bonferroni's *post hoc* test p<0.0001). Black and white bars represent the dark and light periods. All the quantitative data were subjected to Cosinor analysis to test for the presence or absence of 24-h rhythmicity (see Table S1, Figure S2).

What triggers this early increase in circadian cell proliferation? Does it represent a general response to mechanical trauma or is it specifically linked with the loss of a section of the fin? To distinguish between these possibilities, we assayed the number of BrdU-positive nuclei and mitotic cells following gentle mechanical abrasion of the surface of the fin in the absence of amputation. We chose to abrade only half of the caudal fin from each fish so that we could use the remaining half fin as a non-injured control. The fish were maintained under LD cycles and fins were assayed between 24 and 48 hours following abrasion. Significant increases in the number of BrdU-positive nuclei (two-way ANOVA p<0.0001) and in pH3 ser10 levels (two-way ANOVA p<0.0001) were observed in the abraded fin regions compared with the non-abraded controls (Figure 6). Furthermore, clock regulation of S-phase as well as of M-phase was evident in the abraded fin sections (Figure 6, S2 and Table S1).

Thus, these results indicate that mechanical trauma alone is sufficient to trigger a local increase in circadian cell proliferation.

### Circadian cell proliferation contributes to the generation of new epidermal layers

**Figure 7:**
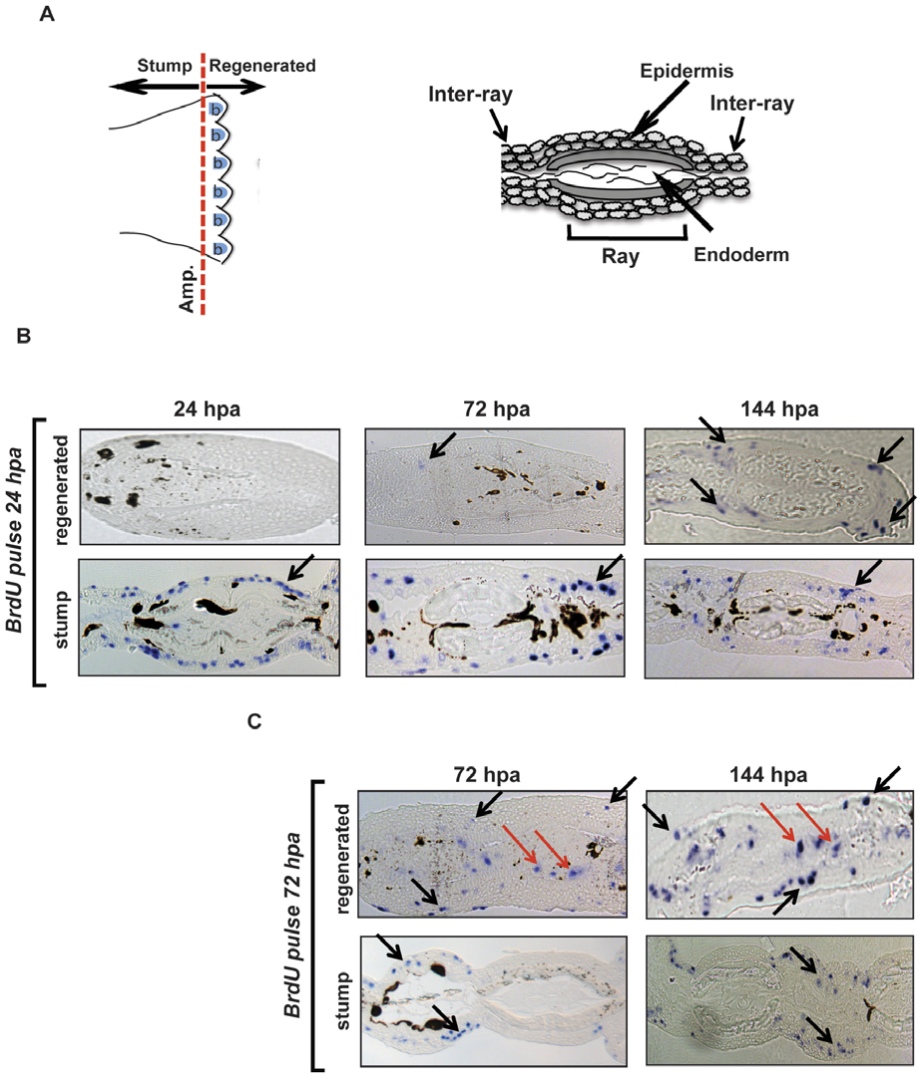
Early proliferating cells contribute to the formation of the new epidermis. (A) Left section: Schematic cartoon of an adult zebrafish caudal fin where the amputation site is indicated (Amp.) and the location of the stump and blastema (b) regions is defined. Right section: Schematic diagram of a transverse section through the zebrafish adult caudal fin. The identity of the principal structures is indicated. (B) Transverse sections of fins that 24 hours following amputation were labeled for 15 minutes with BrdU and then sampled at 24, 72 and 144 hpa. Histological sections through the tip of the new regenerating fin tissue (regenerated) and through the “original” portion of the fin (stump) are represented. Representative blue stained BrdU positive nuclei are indicated by black arrows and are predominantly restricted to the epidermal layers of the stump at all time points and in the regenerated epidermis at 72–144 hpa. (C) Sections from a comparable experiment to that presented in panel B, except that the 15 minutes BrdU labeling period was performed 72 hours after amputation. BrdU positive nuclei are visible in both epidermis (black arrows) and in the blastema region (red arrows) at all time points in the regenerating tissue.

What is the biological significance of the increase in proliferating fin cells that accompanies injury and precedes fin regeneration? What is the contribution of these proliferating cells to the subsequent fin regrowth? In order to specifically track the circadian regulated cells, we transiently labeled (15 minutes) the fin tissue at 24 hpa using BrdU. We then tracked the labeled cells over a period of 6 days in histological cross sections through the fins (Figure 7). As predicted from our initial observations, at 24 hpa, epidermal cells represent the predominant cell population that is labeled with BrdU. These BrdU-positive cells are located proximal to the body with respect to the amputation plane (the stump) but not in the region of the apical epidermal cap (Figure 7B). Subsequently, at 72 and 144 hpa, when the blastema is fully formed, the labeled cells are still visible within the epidermal layer in the original stump tissue. Importantly, at these later timepoints, epidermal cells but not blastema cells are labeled at the growing edge of the new regenerating fin tissue (Figure 7B). In contrast, in fish where the short pulse of BrdU labeling was performed at 72 hpa (Figure 7C), both epidermal and blastema cells are clearly labeled at both later timepoints at the growing edge of the regenerating tissue (Figure 7C). Together, these results indicate that during regeneration, the circadian clock-controlled proliferating epidermal cells contribute to the formation of the new epidermal layers. These cells do not contribute to the formation of the blastema or its subsequent maintenance.

### The timing of injury dictates the kinetics of epidermal cell proliferation

**Figure 8:**
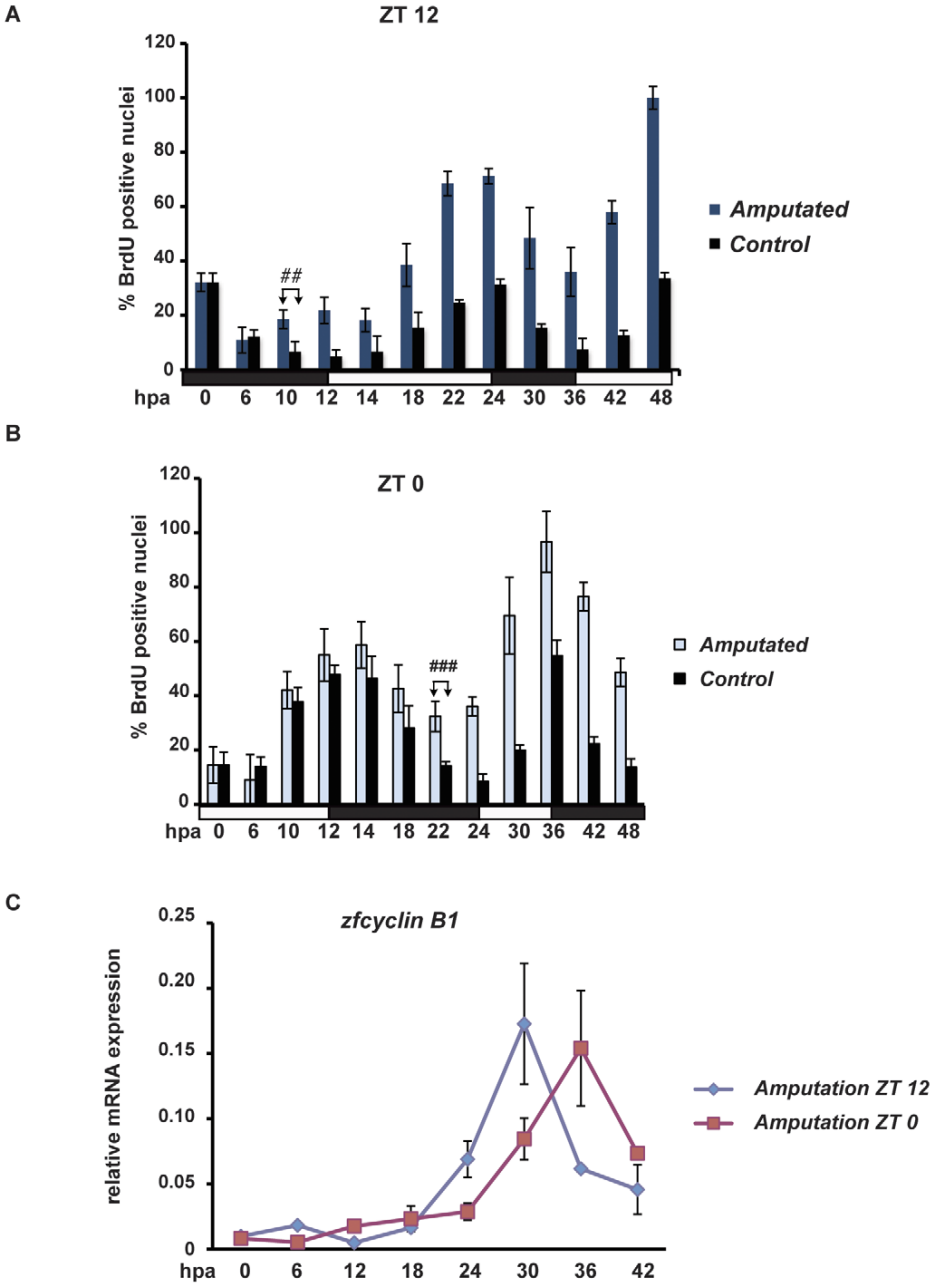
Time of amputation defines kinetics of increased epithelial cell proliferation. (A, B) BrdU incorporation in caudal fins from fish maintained under LD cycles and amputated at the end of the light period (A, ZT12, dark blue bars) or at the end of the dark period (B, ZT0, light blue bars). Results from non-amputated control fish are plotted in both panels (black bars, A and B). In both the panels, on the Y-axis is plotted the % of BrdU positive nuclei with respect to the largest value (A, 48 hpa; B, 36 hpa). A significant increase in cell proliferation is evident sooner in fish amputated at ZT12 (A, 10–12 hpa) compared with fish amputated at ZT0 (B, 22 hpa). Each time point represents the mean value +/− SEM calculated for a minimum of n = 6 fish. In both panels, the first time point showing a significant difference from the control is indicated by the symbol “#” and a bracket. Black and white bars indicate dark and light periods. (C) Levels of *zfcyclin B1* mRNA expression following amputation either at ZT0 (red trace) or ZT12 (blue trace).

Increased cell proliferation in the fin epidermis upon injury displays strong circadian rhythmicity. Thus, does the time of day when the fin is amputated influence the kinetics of increasing cell proliferation? To tackle this question, we compared cell proliferation in fins amputated at two different times of day: at lights on (ZT 0) and at lights off (ZT 12) (Figure 8). Specifically, we compared the increase in BrdU-positive nuclei during the first 48 hpa. We observed a significant difference in the kinetics of the increase in S-phase cells between the two sets of fins compared with the control sets of non-amputated fins (three-way ANOVA, p<0,0001) (compare Figure 8A and B). As a consequence, we observed a significant increase in the number of BrdU-positive nuclei in ZT0 amputated fins 10 hours later (22 hpa) than in the ZT12 amputated fins (10–12 hpa) compared with the respective non-amputated control fins (Figure 8B and 8A respectively). This is consistent with the constraint of the timing of the circadian cycle observed in normal fins (see Figure 2). These data are supported by a delay in the increase of *zfcyclin B1* mRNA expression in fins amputated at ZT0 relative to ZT12 (two-way ANOVA, p<0.0001) (Figure 8C). Thus, as a consequence of the circadian clock regulation, the length of the delay between the injury and the increase in epidermal cell proliferation is not constant. It depends upon the time of day when the amputation occurs.

## Discussion

The correct timing of cell proliferation is of central importance for tissue regeneration. In the case of the regenerating fin to date, most attention has focused on the contribution of blastema cells to this process. Furthermore, interactions between blastema cells and the overlying epidermal cells have been demonstrated to play important roles in the regenerative process. We have studied the timing of cell proliferation both in the blastema and the epidermis of the regenerating zebrafish fin. Previous studies of cell proliferation during fin regeneration in adult fish have frequently relied upon staining for S-phase positive nuclei after a relatively long period of incubation with BrdU. Here, we have employed much shorter S-phase labeling periods (15 minutes), enabling us to visualize at high temporal resolution the precise kinetics of cell proliferation.

The timing of the cell cycle represents a key clock output in most species. Circadian rhythms of S-phase and M-phase have been documented in many tissues in vertebrates, in plants as well as in unicellular organisms [28], [29], [31]. We demonstrate that zebrafish retain day-night rhythms of cell cycle from the larval to adult stages suggesting a key role for this timing mechanism in the normal growth and development of the fish. The persistence of these rhythms during the first few days under constant conditions, clearly points to control by the circadian clock. Many studies have implicated circadian clock regulation of key cell cycle control genes, for example *wee1* and *p21*. Consistently, we have observed rhythmic expression of both genes in adult zebrafish caudal fins. It is also conceivable that clock-derived systemic signals may contribute to driving circadian cell cycle rhythms *in vivo*. Indeed, circulating levels of glucocorticoids have been implicated in circadian cell cycle timing in zebrafish [38]. It has been suggested that circadian control of the cell cycle serves as a protective mechanism. This ensures that sensitive phases of the cell cycle such as DNA replication occur at times of day when there is reduced exposure to UV light from the sun [44], [45], [46]. Given that the natural habitats of zebrafish are in shallow bodies of water where fish are likely to be exposed to UV light, the clock control of cell proliferation could indeed represent a relevant protective mechanism particularly for the epidermis. Interestingly, regular pulses of cell proliferation leading to bone formation have been reported during normal fin growth in the zebrafish [41]. Given the importance of crosstalk between epidermal and blastema cells in directing cell differentiation in the regenerating fin, it is tempting to speculate that the epidermal cell cycle rhythms may also contribute to growth rate.

Our data also shed light on basic aspects of skin function in adult teleosts. The skin in larval and adult zebrafish is characterized by relatively high levels of proliferating cells. The destiny of these cells remains unclear, given that there is no counterpart of the outer cornified layer present in mammals. One could speculate that mechanical abrasion associated with normal swimming behavior will inevitably lead to continuous cell exfoliation that must be counteracted by sustained cell proliferation. Our finding that gentle mechanical abrasion also leads to a general increase in epidermal cell proliferation would tend to support this notion. However, in order to confirm this hypothesis, more systematic studies tracking the fate of these proliferative cells will be necessary. Importantly, we have also demonstrated that epidermal cell proliferation is strongly regulated by the circadian clock. This leads to different kinetics of increased cell proliferation after injury according to the time of day. There is a consequent restriction of S phase and M phase to the light/dark transition or dark period respectively. This finding could have important medical implications suggesting that proliferation and regeneration are greater when tissue damage occurs at a certain time of day. The clinical ramifications of this could possibly involve guiding the timing of surgery in order to speed healing and recovery.

In sharp contrast, we failed to detect circadian rhythms of proliferation in the blastema. However, we did observe daily oscillations of circadian clock gene expression in the blastema region. Together, these findings raise a very basic question. Why should cell proliferation in certain cell types be strongly clock regulated and not in others? One could speculate that it may be more critical for proliferating epidermal cells than for blastema cells to avoid the damaging effects of sunlight. Alternatively, it may be advantageous to restrict cell proliferation on the surface of the skin to the nighttime when the wear and tear on the fin structure would be reduced. The maintenance of uninterrupted cell proliferation in the blastema may be essential to ensure the most rapid repair of the fin structure possible. Given the strong circadian regulation of epidermal cell behaviour described in this work and the importance of epidermal – mesenchymal cell interactions in the regeneration process, it is tempting to speculate that the circadian clock mechanism may contribute at various levels to the timing of fin regeneration.

## Materials and Methods

### Fish care, treatment and ethical statements

The zebrafish Tuebingen strain were raised and bred according to standard procedures [47] in a re-circulating water system under 14 hours light and 10 hours dark cycles at 28°C and fed twice per day.

Before each experiment adult zebrafish (6–12 months of age) were adapted for a minimum of 15 days at a constant 28°C under a 12-h light: 12-h dark cycle (LD) (light intensity, 20 µW/cm^2^), or in constant darkness (DD). The fish were fed twice randomly on a daily basis using an automatic feeder to ensure that the entrainment of the clock was due only to the light cycle and not to the feeding behaviour. The caudal fins were amputated using razor blades following anesthesia with 0.02% MS222 (3-aminobenzoate methanesulfonic acid, Sigma Aldrich). In the regeneration experiments, the control fish (non-amputated) were anesthetized and handled in the same way and at the same times as the amputated fish. The mechanical abrasion of the fin was performed by gentle rubbing half of the fin surface using the thin edge of a MultiFlex gel loading tip (Peqlab) without breaking the structure of the fin. The remaining, non-abraded half of the fin served as an internal control.

### Ethics statement

All zebrafish husbandry and experimental procedures were performed in accordance with the German animal protection standards (Animal Protection Law, BGBl. I, 1934 (2010)) and were approved by the Local Government of Baden-Württemberg, Regierungspräsidium Karlsruhe, Germany (License no. regeneration project (fin clips): Az.: 35-9185.82/A-47/08. BrdU treatments Az.: 35-9185.81/G-20/06 and general license for fish maintenance and breeding: Az.: 35-9185.64)

### BrdU assay

Fish were incubated for 15 minutes in 10 mM BrdU fish water solution at different time points during the day-night cycle and then the caudal fins were amputated and immediately fixed overnight in Carnoy's solution (60% ethanol, 30% chloroform, 10% acetic acid). Staining for BrdU incorporation was carried out as described previously [31] with some modifications. In particular, after a sequential series of rehydration steps in 100%, 66% and 33% methanol in PBTX (1× PBS, 0,3%Triton X100), the fins were incubated for 30 minutes in 2 N HCl in PBTX and then for a minimum of 4 hours in prehybridization solution (PBTX, 0.25%BSA). After overnight labeling at 4°C with the primary anti-BrdU mouse antibody (1∶50) (ICN) followed by the secondary horse anti-mouse alkaline phosphatase-conjugated (AP) antibody (1∶500) (Vector), the BrdU positive nuclei were visualized using an alkaline phosphatase assay method as previously described [47].

The BrdU positive nuclei were counted following photography using a Zeiss stereomicroscope Stemi SV11 with 3.2× magnification and Zeiss Axiocamera MRC. In each fin picture, two separate rectangular areas of 500×200 pixels were marked corresponding to an actual size of 1 mm×0,4 mm. The mid-longitudinal line and the bifurcation point of the fin were used as reference points. The two rectangles were draw at approximately 0.4 mm from the mid-line and 0.5 mm from the bifurcation point of the fin. Each rectangular area covers circa 1 fin ray and its two lateral inter-rays. The number of positive nuclei was calculated as the sum of the BrdU nuclei counted in the two areas. Each experiment was performed in triplicate. For each time point (minimum 4 fins per experiment) the mean number of positive nuclei was plotted against the ZT (zeitgeber) or CT (circadian) time and statistical analysis was performed. The transversal fin sections were prepared as previously described [48]. The level of BrdU positive nuclei in the blastema region was quantified using Scion Image software (NIH, http://rsb.info.nih.gov/nih-image/).

#### Statistical analysis

Data were analyzed by one-way analysis of variance ANOVA followed by Bonferroni's multiple comparison test using the GraphPad Prism 4.0 for Windows (Graph Pad Software, http://www.graphpad.com). Two-way and three-way ANOVA were performed using SPSS v 16.0 for Windows (IBM, USA). Cosinor analyses were performed using COSINOR v3.0.2 software (Prof. Antoni Diez-Noguera, University of Barcelona). All the results were expressed as means +/− SEM. p<0.05 was considered statistically significant. In each figure, p<0.05, p<0.001 and p<0.0001 are represented by * or #, ** or ## and *** or ### respectively.

#### RNA analysis

Total RNA samples were extracted using Trizol RNA isolation reagent (GIBCO-BRL) according to the manufacturer's instructions. Total RNA was reverse-transcribed into cDNA by using Superscript III Reverse Transcriptase (Invitrogen) with a mix of oligo dT and random primers. Quantitative Real Time RT-PCR analysis was performed using a StepOnePlus Real-Time RT-PCR System (Applied Biosystems) and SYBR Green I fluorescent dye (Quiagen). Relative expression levels were normalized using *zf*β-*actin*. The relative levels of each mRNA were calculated using the 2-ΔΔCT method. For each gene the primer sequences used for quantitative Real Time RT-PCR are described in Table S2.

#### Western blotting

Protein extracts were prepared by homogenizing samples in Laemmli buffer including a cocktail of phosphatase inhibitors 2 (Sigma). The samples were electrophoresed on a SDS polyacrylamide gel and transferred to a Hybond-P membrane (Amersham). Binding of the antibodies for Histone H3 and Phospho-histone H3 Ser10 (Cell Signaling) was visualized using the ECL detection system (Amersham Biosciences). Autoradiographic images were quantified with the aid of Scion Image software (NIH, http://rsb.info.nih.gov/nih-image/). Statistical analysis was performed with the aid of GraphPad PRISM 4.0 software.

#### Primary culture and transfection of fin-derived cells

Fin clips from adult zebrafish caudal fins were trypsinized and seeded into 24-well plates in L15 medium supplemented with 20% foetal calf serum, penicillin and streptomycin. The primary culture was incubated for 1 week at 25°C using standard methods for zebrafish cell culture described elsewhere [49]. After 1 week, the cells were transiently transfected with the clock-regulated reporter construct *zfPer1b-luc* [25] using FuGene HD reagent according to the manufacturers recommendations (Roche). Bioluminescence was assayed *in vivo* using an Envision multilabel counter (Perkin Elmer) under various lighting conditions.

## Supporting Information

Figure S1 **Schematic representation of the structure of the zebrafish fin.** (**A**) Diagram of a transverse section through the zebrafish adult caudal fin. The identity of the principal structures is indicated. (**B**) Diagram of a longitudinal section through a fin ray following amputation at a stage when the blastema (blue cells) is fully formed. The original site of amputation (dotted red line) as well as the orientation and the principle structures are indicated.
(TIF)

Figure S2 **Acrophase analysis.** Acrophase plot for all significant 24-h rhythms analysed in this study (Cosinor, p<0.05). The acrophase and fiducial limits (set at 95%), calculated by Cosinor analysis for each experiment, are indicated by a symbol and lateral bars, respectively. Empty circles, black circles and empty triangles indicate data from LD, DD and LL lighting conditions respectively. Black and white bars, as well as grey and white background, indicate the dark and light periods of the lighting regimes. On the X-axis the time is indicated either as zeitgeber time (ZT) or circadian time (CT).
(TIF)

Figure S3 **Loss of BrdU incorporation rhythms under constant conditions.** (A) Representative image of BrdU staining of zebrafish caudal fin under LD conditions at ZT 9. BrdU positive nuclei (blue spots) in the inter-ray regions are indicated by black arrows. Red arrows indicate the few BrdU positive nuclei in the ray regions of the fin. (B) BrdU incorporation assays of fins sampled from fish maintained for 15 days under constant darkness (DD, black bars) or constant light (LL, white bars) and sampled at 6 hourly intervals during one subsequent 24 hours cycle (plotted as CT times). On the Y-axis is plotted the % of BrdU positive nuclei with respect to the largest value (DD, CT 9 and LL, CT15). Each time point represents the mean value +/− SEM calculated for a minimum of n = 6 fish. The data were subjected to Cosinor analysis to test for the absence of 24-h rhythmicity (see Table S1).
(TIF)

Figure S4 **High amplitude circadian epithelial cell cycle rhythms persist during fin re-growth.** Representative fin segments stained for BrdU incorporation at four zeitgeber times (ZT) distributed through one 24 hours cycle, starting 7 days post amputation (7 dpa). Below, quantification of the level of BrdU staining in fins amputated 7 days previously (blue bars) compared with non-amputated control fins (black bars). On the Y-axis is plotted the % of BrdU positive nuclei with respect to the largest value (ZT 9, 7 dpa). Each time point represents the mean value +/− SEM calculated for a total of n = 6 fins. The result of statistical analysis of the peak and trough values for the amputated fins is indicated by asterisks (Bonferroni's *post hoc* test p<0.0001) and horizontal “brackets” above the graph. Furthermore, statistically significant differences observed at each time point between the amputated and non-amputated fins are indicated for simplicity, by the symbol “#” and a bracket above only the first time point (ZT3) (Bonferroni's *post hoc* test p<0.001). White and black bars denote the light and dark periods respectively. The data were subjected to Cosinor analysis to test for the absence or presence of 24-h rhythmicity (see Table S1, Figure S2).
(TIF)

Table S1 **Cosinor analysis.** Summary of the significance values from the Cosinor analysis used to test for the presence or absence of 24-h rhythmicity. The significance threshold was set at α = 0.05. For *zfwee1*, the Cosinor analysis concludes no significant rhythmicity. However, a daily change in expression is evident with very sharp peaks although the kinetics do not correspond to a cosine function curve.
(DOC)

Table S2 **PCR primers.** Summary of the sequences of forward (F) and reverse (R) PCR primers used for quantitative RT-PCR analysis.
(DOC)
