# Supplementary figures and images for: Circadian Timing of Injury-Induced Cell Proliferation in Zebrafish
Source: PLoS One. 2012 Mar 29;7(3):e34203. doi: 10.1371/journal.pone.0034203 (PMC3315524; doi:10.1371/journal.pone.0034203)

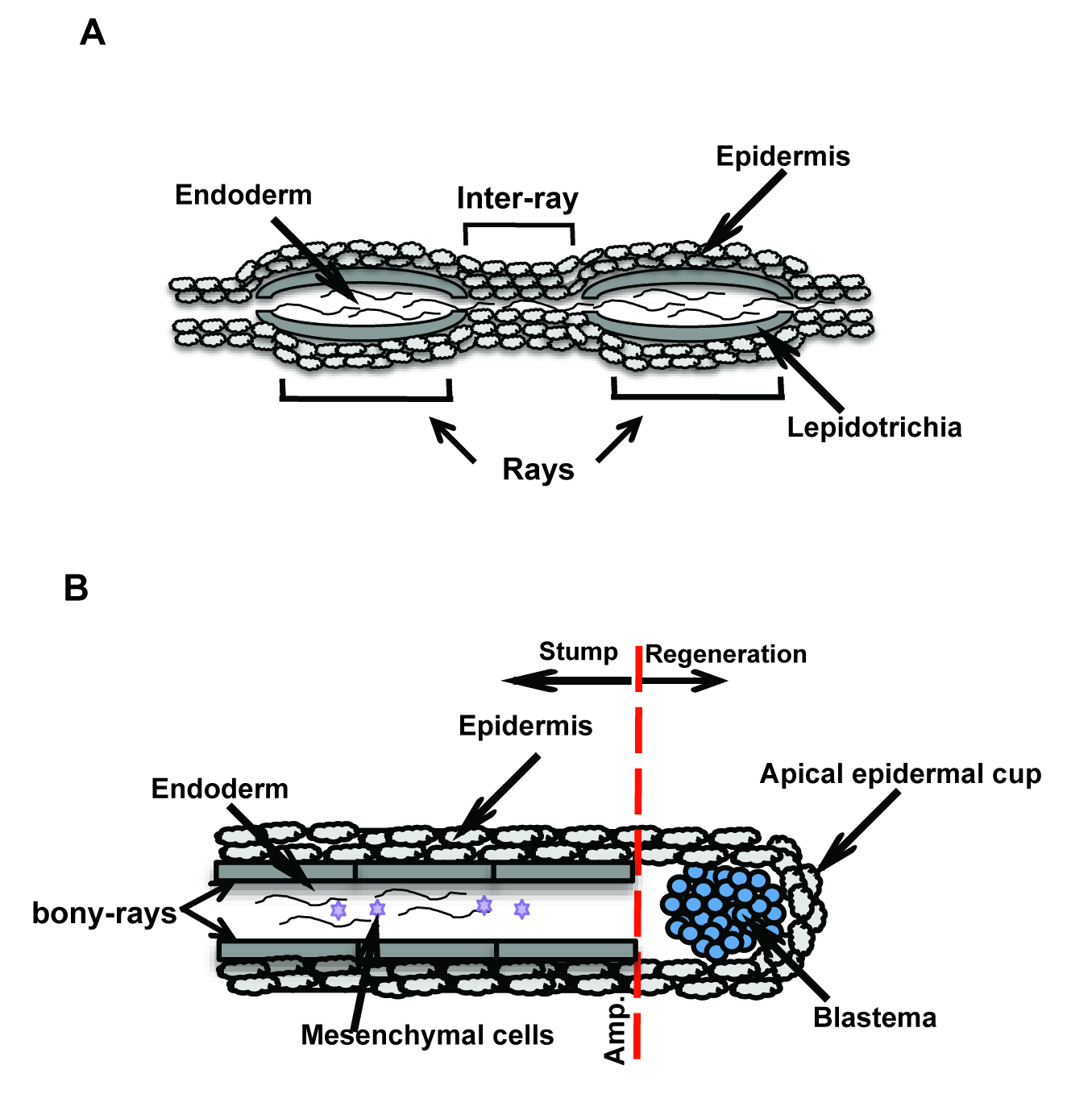

Supplement: Figure S1 — Schematic representation of the structure of the zebrafish fin. (A) Diagram of a transverse section through the zebrafish adult caudal fin. The identity of the principal structures is indicated. (B) Diagram of a longitudinal section through a fin ray following amputation at a stage when the blastema (blue cells) is fully formed. The original site of amputation (dotted red line) as well as the orientation and the principle structures are indicated. (TIF) [file pone.0034203.s001.tif]

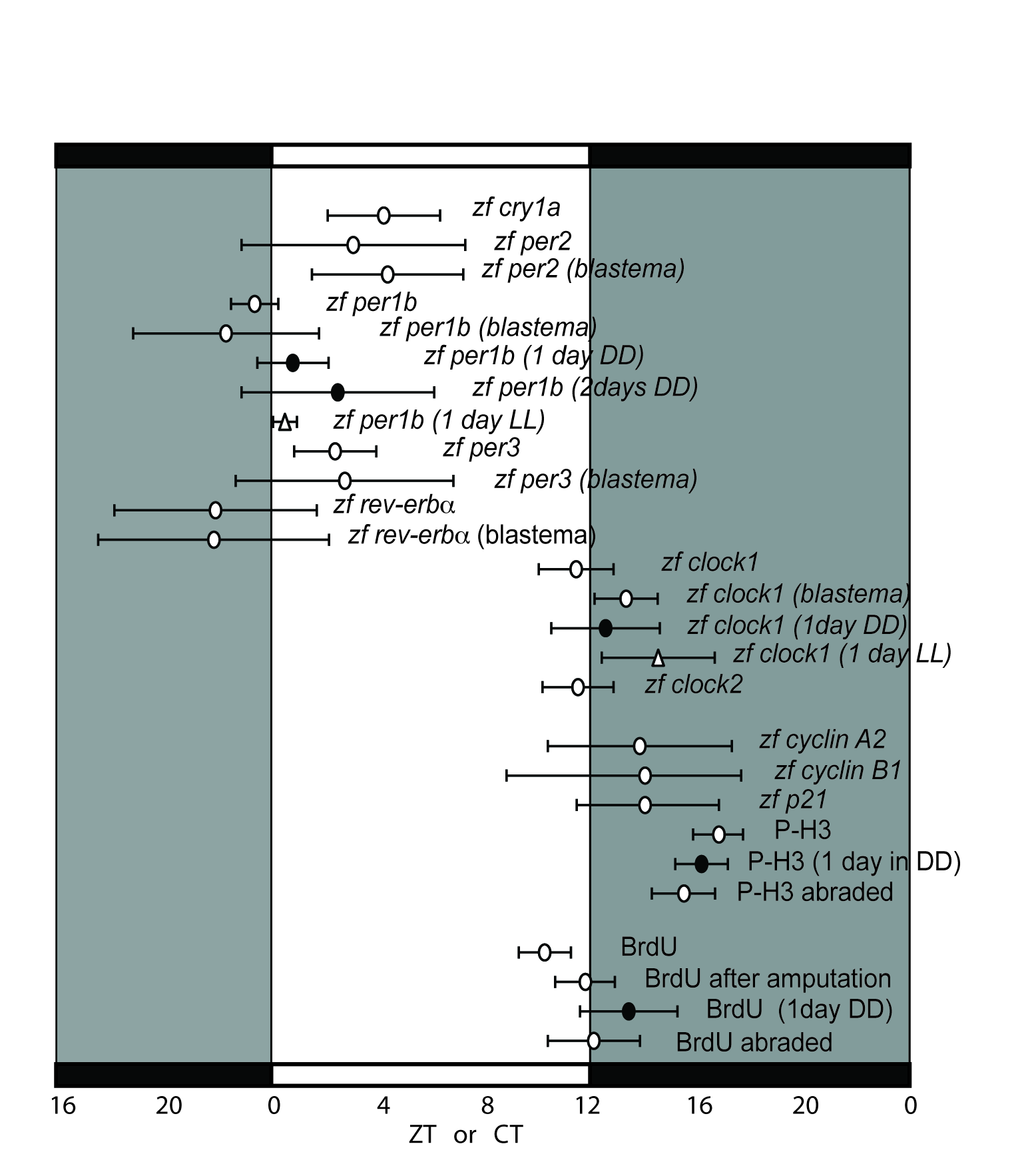

Supplement: Figure S2 — Acrophase analysis. Acrophase plot for all significant 24-h rhythms analysed in this study (Cosinor, p<0.05). The acrophase and fiducial limits (set at 95%), calculated by Cosinor analysis for each experiment, are indicated by a symbol and lateral bars, respectively. Empty circles, black circles and empty triangles indicate data from LD, DD and LL lighting conditions respectively. Black and white bars, as well as grey and white background, indicate the dark and light periods of the lighting regimes. On the X-axis the time is indicated either as zeitgeber time (ZT) or circadian time (CT). (TIF) [file pone.0034203.s002.tif]

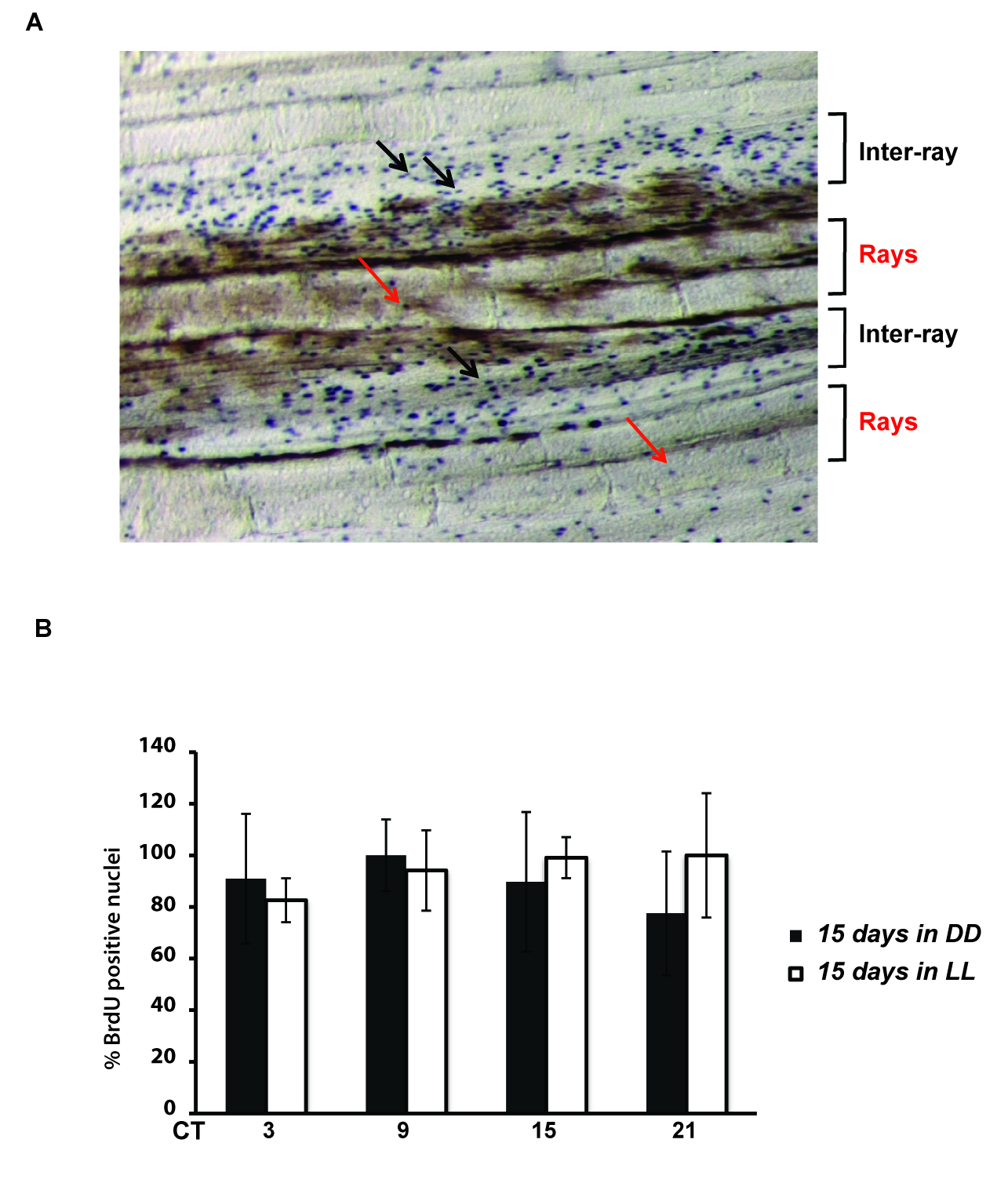

Supplement: Figure S3 — Loss of BrdU incorporation rhythms under constant conditions. (A) Representative image of BrdU staining of zebrafish caudal fin under LD conditions at ZT 9. BrdU positive nuclei (blue spots) in the inter-ray regions are indicated by black arrows. Red arrows indicate the few BrdU positive nuclei in the ray regions of the fin. (B) BrdU incorporation assays of fins sampled from fish maintained for 15 days under constant darkness (DD, black bars) or constant light (LL, white bars) and sampled at 6 hourly intervals during one subsequent 24 hours cycle (plotted as CT times). On the Y-axis is plotted the % of BrdU positive nuclei with respect to the largest value (DD, CT 9 and LL, CT15). Each time point represents the mean value +/− SEM calculated for a minimum of n = 6 fish. The data were subjected to Cosinor analysis to test for the absence of 24-h rhythmicity (see Table S1). (TIF) [file pone.0034203.s003.tif]

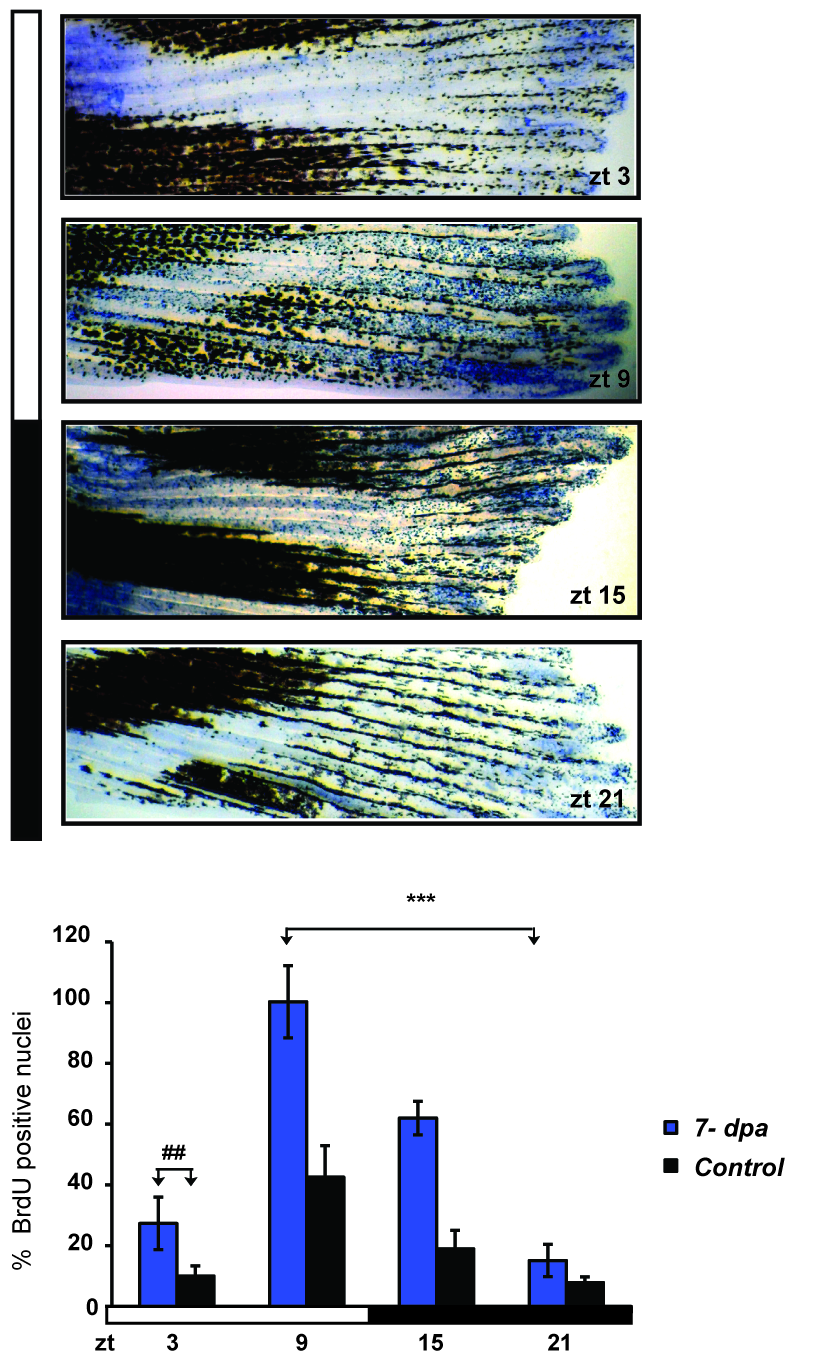

Supplement: Figure S4 — High amplitude circadian epithelial cell cycle rhythms persist during fin re-growth. Representative fin segments stained for BrdU incorporation at four zeitgeber times (ZT) distributed through one 24 hours cycle, starting 7 days post amputation (7 dpa). Below, quantification of the level of BrdU staining in fins amputated 7 days previously (blue bars) compared with non-amputated control fins (black bars). On the Y-axis is plotted the % of BrdU positive nuclei with respect to the largest value (ZT 9, 7 dpa). Each time point represents the mean value +/− SEM calculated for a total of n = 6 fins. The result of statistical analysis of the peak and trough values for the amputated fins is indicated by asterisks (Bonferroni's post hoc test p<0.0001) and horizontal “brackets” above the graph. Furthermore, statistically significant differences observed at each time point between the amputated and non-amputated fins are indicated for simplicity, by the symbol “#” and a bracket above only the first time point (ZT3) (Bonferroni's post hoc test p<0.001). White and black bars denote the light and dark periods respectively. The data were subjected to Cosinor analysis to test for the absence or presence of 24-h rhythmicity (see Table S1, Figure S2). (TIF) [file pone.0034203.s004.tif]
