# Supplementary material for: Circadian Timing of Injury-Induced Cell Proliferation in Zebrafish
Source: PLoS One. 2012 Mar 29;7(3):e34203. doi: 10.1371/journal.pone.0034203 (PMC3315524; doi:10.1371/journal.pone.0034203)
Supplement: Table S1 — Cosinor analysis. Summary of the significance values from the Cosinor analysis used to test for the presence or absence of 24-h rhythmicity. The significance threshold was set at α = 0.05. For zfwee1, the Cosinor analysis concludes no significant rhythmicity. However, a daily change in expression is evident with very sharp peaks although the kinetics do not correspond to a cosine function curve. (DOC) [file pone.0034203.s005.doc]

**Table S1.**

**Cosinor analysis**

| **Data** | **Lighting condition** | **Cosinor significance** |
| --- | --- | --- |
| *zfclock2* | LD | p<0.001 |
| *zfcry1a* | LD | p<0.005 |
| *zfper2* | LD | p<0.05 |
| *zfper2 blastema* | LD | p<0.01 |
| *zfper3* | LD | p=0.001 |
| *zfper3 blastema* | LD | p<0.05 |
| *zfper1b* | LD | p<0.0001 |
| *zfper1b blastema* | LD | p<0.05 |
| *zfper1b* | DD 1 day | p<0.001 |
| *zfper1b* | DD 2 days | p<0.05 |
| *zfper1b* | DD 15 days | p= 0.31 (n.s) |
| *zfper1b* | LL 1 day | p<0.00001 |
| *zfclock1* | LD | p<0.0005 |
| *zfclock1 blastema* | LD | p<0.0005 |
| *zfclock1* | DD 1 day | p<0.001 |
| *zfclock1* | LL 1 day | p<0.005 |
| *zfrev-erbα* | LD | p<0.05 |
| *zfrev-erb*α* *blastema | LD | p<0.05 |
| *zfcyclin A2* | LD | p<0.05 |
| *zfcyclin b1* | LD | p<0.05 |
| *zfp21* | LD | p<0.001 |
| *zfwee1* | LD | p= 0.077 (n.s) |
| P-H3 | LD | p<0.00001 |
| P-H3 | DD 1 day | p<0.00001 |
| P-H3 abraded | LD | p<0.05 |
| BrdU | LD | p<0.00001 |
| BrdU | DD 1 day | p<0.0005 |
| BrdU | DD 15days | p= 0.718 (n.s) |
| BrdU | LL 15days | p= 0.319 (n.s) |
| BrdU 18-60hpa | LD | p<0.00001 |
| BrdU blastema | LD | P=0.72 (n.s) |
| BrdU 7dpa | LD | p<0.0005 |
| BrdU abraded | LD | p<0.0005 |
