# Supplementary material for: Circadian Timing of Injury-Induced Cell Proliferation in Zebrafish
Source: PLoS One. 2012 Mar 29;7(3):e34203. doi: 10.1371/journal.pone.0034203 (PMC3315524; doi:10.1371/journal.pone.0034203)
Supplement: Table S2 — PCR primers. Summary of the sequences of forward (F) and reverse (R) PCR primers used for quantitative RT-PCR analysis. (DOC) [file pone.0034203.s006.doc]

**Table S2.**

**PCR primers**

| **Gene** | **Quantitative RT-PCR Primers** |
| --- | --- |
| *zfper2* | F: ATGTCGATGGCTTTAGGCAG  R: CGAGACATCCAGAAGGTGCT |
| *zfper1b* | F: CCGTCAGTTTCGCTTTTCTC  R. ATGTGCAGGCTGTAGATCCC |
| *zfcry1a* | F: TCCGCTGTGTGTACATCCTC  R: CAAACACTGCAGCAAAAACC |
| *zfclock1* | F: CTGGAGGATCAGCTGGGTAG  R: CACACACAGGCACAGACACA |
| *zfclock2* | F: AGAACTGCTGAGGCTGCTGT  R: TAACGTTGTGCTAGTCCCCT |
| *zfper3* | F: CCTTCGGAAACAAGCCATTA  R: CCGCCTCCATAGAAAATCAA |
| *zf rev-erb*α | F: GGACAAGCCAGCAGAATCTC  R: CCTGAAAAACATCAGCAGCA |
| *zf*β*-actin* | F: GCCTGACGGACAGGTCAT  R: ACCGCAAGATTCCATACCC |
| *zfcyclin A2 (ccna2)* | F: TAGATTGCGATCCCTTCCTC  R: CCTGTTGAGCGTGTTGAGAA |
| *zfcyclin b1 (ccnb1)* | F: GGTCCACTACCCTCCCTCTC  R: ATGCTTAGAAAGGCCCTCGT |
| *zfwee1* | F: ACCAGCTGCGTGTAGAGCTT  R: GCTGGACTGGATTGTGGAAC |
| *zfp21* | F: TGACATCAGCGGGTTTACAG  R: TTCTGCTGCTTTTCCTGACA |
| *zfmsxb* | F: GACCCGTTGAAACGACATCT  R: TGTCCCATTCTCTGATGCTG |
